# The Week's Work

**Published:** 1910-01-29

**Authors:** 


### The Week's Work.

### "WHEN WE DEAD AWAKEN."

The Salford Union Workhouse Infirmary at Hope, by the new spirit which has characterised the utterances and action of the Board of Guardians at recent meetings, aptly fits Ibsen's graphic phrase just quoted. When, as the result of a careful inspection, Sir Henry Burdett described this institution as "belonging to the Charles Dickens type, which has far too long been allowed to shock the public conscience by the inefficiency and incapacity of the Board of Guardians," Mr. Bratherton, the chairman of the Infirmary Committee, characterised these criticisms as vile and wicked aspersions, and failed to see that there could be a shadow of truth in them. The Chairman of the Board contended there was not the slightest warrant for so terrible a charge, and that he had never read such a misleading, villainous, and untruthful charge, which he believed to be an absolute falsehood. At the meeting of the Board of Guardians on January 21 Mrs. Shutt called attention to the fact that 45 children suffering from ophthalmia were under the care of the Board in sheds at Hope Hospital. Many of the children had been there for years, and she was convinced that if an eye specialist had been appointed years ago much suffering and permanent disfigurement would have been avoided. The old system, presumably of the Board, had had a long trial, and had been a miserable failure. Mr. Bratherton described the place assigned to the children as horrible sheds, miserable sheds, to which he wished someone would perform the sacred duty of applying a match and burning the lot completely. The first duty of the Board was to deal with these sheds, which were in a miserable, horrid, damp condition. Other members of the Board declared that the sheds were structurally so bad that "the beds had to be pulled away from the walls to prevent them from getting wet"; and that "on one Sunday the children in the sheds were shivering in their beds whilst the attendants were engaged in swilling out the water." Such a state of things, and the admissions of Mr. Bratherton and his *confrères* are in striking contrast to the resolution of the Board which we published on page 346 of The Hospital of December 18, in which the Board claimed that Sir Henry Burdett's criticism cast a serious reflection and unmerited aspersion on the institution and those responsible for its upkeep and management. The Board have consequently spoken with two voices within the last two months, and we welcome the second voice in which they admit, in language more severe even than that used by Sir Henry Burdett, the conditions under which the children have been housed and treated. We are glad to note that the Board determined to appoint an eye specialist, and also, with the approval of the Local Government Board, a medical superintendent, who is to devote his whole time to the work and to have full control of the Hope Hospital. One of the earliest results of this appointment will doubtless be the demolition and destruction of the sheds in which some of the children are at present housed, and the thorough reorganisation and improvement of the whole conditions under which children are treated and retained in other portions of this infirmary. We look forward to the time when the whole of this institution will be reorganised and brought up to date, so as to make it thoroughly efficient from top to bottom.

### HOME CHALETS FOR TUBERCULAR PATIENTS.

In a note under this heading in The Hospital of January 15 we omitted to state that Mr. Stanley Bates occupied his garden chalet for one year without ever being indoors for more than ten minutes in any twenty-four hours. We inadvertently made it appear that he had so resided for three years, and Mr. Bates thinks that so long a residence might lead to a measure of incredulity on the part of the reader, though we have known cases where residence in these chalets has lasted for a longer period than three years, with increasing comfort and benefit to the occupants. The point we wished to drive home was that home chalets should be supplied by local authorities to the poorer ratepayers who have gardens directly a member of their household is afflicted with tubercular disease. If this treatment can be resorted to at the commencement of the attack or immediately afterwards, then there is hope that in suitable cases, at any rate, patients so treated may have the good fortune of Mr. Bates, and be able to resume their ordinary vocations at the end of twelve months.

### HOSPITAL FINANCE IN WALES.

We publish in another column a letter from the General Superintendent of the Cardiff Infirmary in reference to a discussion which took place at a meeting of the committee of that institution, as reported in our columns on January 22. Lord Aberdare, the treasurer, in calling attention to the bank overdraft of £24,000, made the rather remarkable statement that so long as this overdraft was kept below £20,000 he was bound to be satisfied, but £24,000 caused him to fear the consequences. We are not accustomed in London to finance the voluntary hospitals with overdrafts of these magnificent proportions. Mr. Rea, in his letter, explains that the Board is now working most strenuously and successfully with the single object of increasing the income. This has been so successful that within the last twelve months £40,000 has been added to investment account and the subscription list has been increased by £1,500 a year. This policy, Mr. Rea explains, has been adopted as the result of local circumstances and experience, and is well understood and approved of by the wealthy friends of the hospital. May we conclude from this explanation that the bankers treat the Cardiff Infirmary with special generosity by not charging them interest on the overdraft of £24,000? We assume some such explanation, for, as a matter of business, with a bank rate at 5 per cent., we do not suppose Lord Aberdare and his Finance Committee would have sanctioned an investment of £40,000 in securities at a small rate of interest before the debt to the bankers had been liquidated, and the heavy charge for interest, if, indeed, interest is charged, had been removed from the account. In any case, the situation presented must have special interest to every hospital manager, and we should very much like to know what are the actual facts on the point just mentioned.

### METROPOLITAN HOSPITAL SUNDAY FUND.

The *National Church* has again made an analysis of the collections in the churches and chapels of the metropolis on Hospital Sunday in London in 1909. The total contributions through these collections was over £1,100 less than the amount received in 1908, whilst the sum received through the Church of England is upwards of 75 per cent. of the total collections, as the following complete list shows:—

| | | | |
|---|---|---|---|
| Church of England | £30,928 | 8 | 6 |
| Congregationalists | 1,626 | 10 | 10 |
| Jews | 1,439 | 11 | 0 |
| Wesleyans | 993 | 6 | 7 |
| Presbyterians | 989 | 19 | 7 |
| Baptists | 809 | 9 | 5 |
| Roman Catholics | 653 | 6 | 7 |
| Unitarians | 365 | 6 | 0 |
| Church of Scotland | 250 | 11 | 1 |
| Theistic Church | 175 | 0 | 0 |
| German Lutherans | 95 | 0 | 5 |
| Greek Church | 89 | 9 | 1 |
| Society of Friends | 87 | 0 | 3 |
| Catholic Apostolic | 85 | 11 | 9 |
| Foreign Protestants | 61 | 16 | 9 |
| Methodists (Primitive) | 55 | 19 | 3 |
| Methodists (Welsh Calvinistic) | 50 | 14 | 10 |
| Methodists (United Free Church) | 19 | 1 | 6 |
| Swedenborgians | 14 | 8 | 1 |
| Reformed Episcopal Church | 13 | 16 | 7 |
| Free Church of Scotland | 8 | 5 | 9 |
| Moravians | 5 | 1 | 9 |
| Various | 300 | 7 | 9 |
| | £39,118 | 3 | 4 |

A sum of £4,326 was received from St. Paul's Cathedral, which consequently heads the list, but the money includes all the donations which were received by the Secretary from the City, so that St. Paul's collection cannot be compared with that of any other place of worship The first congregation on the list is therefore Christ Church, Lancaster Gate, which contributed £1,001. We hope that Bishop Gore's hint, to which we called attention in our issue of November 13 last, p. 169, will be adopted throughout the metropolis and the country in connection with these collections during 1910. Once secure the enforcement of the Bishop's suggestion, and the Metropolitan Hospital Sunday Fund may speedily reach £100,000 a year.

### THE PLANNING OF NEW POOR LAW INFIRMARIES

The Wayland Guardians have decided that the time has arrived for them to erect a new Poor Law Infirmary, and they have accordingly appointed a Sites Committee to select a suitable site for the approval of the Local Government Board. We are glad to know that the Guardians, according to the report, seem practically unanimous that the new infirmary shall be as far as possible a model one, and we hope they will make this possible by appointing two assessors, one an architect, and the other someone who has an intimate knowledge of modern hospitals, to adjudicate on the plans. An alternative course would be for them to seek the aid of such assessors, to whom they would submit their requirements and ask them to prepare pencil drawings, so that the whole of the plans and arrangements may from the first be on the most practical and up-to-date lines. Over and over again, we have shown by reference to existing hospitals of various kinds that money alone will never produce an efficient plan, for the first consideration is not a multitude of sovereigns, but the application of good brains and practical experience to the production of the best possible plan. Unless the Wayland Guardians take special precautions in these directions and resolve to avoid the causes which have produced so many unsatisfactory hospital buildings in the past, they will never succeed in securing a modern, up-to-date Poor Law Infirmary, spend what they may.

### FROM THE PATIENT'S POINT OF VIEW.

In a former paragraph in these columns we laid stress upon the desirability of hospital workers knowing something about the views and impressions of hospital patients, and since then we have received several articles dealing with ward routine and daily hospital life from the point of view of men and women who have been patients in institutional wards and have more or less faithfully and honestly recorded their impressions. One of these is before us—a patient's record, as it appears in the *Freeman's Journal*, of daily life in a Dublin hospital. It is a singularly elucidative and interesting article, and it contains valuable hints to hospital workers. Take, for instance, the question of students' demonstrations. The writer of the article deals in a humorous and genial fashion with the visits of the staff physicians and his crowd of attendant clerks. "The patient," he remarks, "enjoys the situation as much as anyone; he generally doesn't mind being made the subject of the lecture; it gives him an opportunity of hearing all his troubles described in the most scientific medical phraseology. After a time he has the full repertoire off by heart, and, next to being cured, this is the most interesting consummation the average invalid looks for." This is a very sensible view to take of a matter which admits of more than one opinion, and it would be interesting to hear what other patients think about it.

### THE DRUG MARKET.

There has been little to attract especial interest in the drug market during the week, and although a fair amount of business has been transacted there have been few price changes. The General Election has had an unsettling influence on trade generally, and until the political situation is better defined the markets will not assume their normal state. The price of ipecacuanha has advanced by about 9d. per lb. as a result of a large order which has been received from the United States. Santonin is 2s. 6d. per lb. dearer, and the syndicate of the factories in Turkestan appears to have regained complete control of the market; the probability is that the price may be advanced again, but there is nothing to indicate that this will take place in the immediate future. Castor oil is tending dearer. The price of cod-liver oil is maintained, but everything now depends upon the results of the Norwegian fishing, which has just commenced. Although the price of cocaine may be considered comparatively low, there seems a possibility that a slight reduction may be made in the price; but this is not altogether certain.

